# Coordinating cell polarity and cell cycle progression: what can we learn from flies and worms?

**DOI:** 10.1098/rsob.130083

**Published:** 2013-08

**Authors:** Anna Noatynska, Nicolas Tavernier, Monica Gotta, Lionel Pintard

**Affiliations:** 1 Department of Physiology and Metabolism, Faculty of Medicine, University of Geneva, 1 rue Michel Servet, 1211 Geneva, Switzerland; 2 Institut Jacques Monod, CNRS, UMR 7592, Univ Paris Diderot, PRES Sorbonne Paris Cité, 75205 Paris, France

**Keywords:** cell polarity, cell cycle, *Drosophila melanogaster*, *Caenorhabditis elegans*

## Abstract

Spatio-temporal coordination of events during cell division is crucial for animal development. In recent years, emerging data have strengthened the notion that tight coupling of cell cycle progression and cell polarity in dividing cells is crucial for asymmetric cell division and ultimately for metazoan development. Although it is acknowledged that such coupling exists, the molecular mechanisms linking the cell cycle and cell polarity machineries are still under investigation. Key cell cycle regulators control cell polarity, and thus influence cell fate determination and/or differentiation, whereas some factors involved in cell polarity regulate cell cycle timing and proliferation potential. The scope of this review is to discuss the data linking cell polarity and cell cycle progression, and the importance of such coupling for asymmetric cell division. Because studies in model organisms such as *Caenorhabditis elegans* and *Drosophila melanogaster* have started to reveal the molecular mechanisms of this coordination, we will concentrate on these two systems. We review examples of molecular mechanisms suggesting a coupling between cell polarity and cell cycle progression.

## 2. Introduction

An adult human is built from roughly 10^13^ cells, which are all generated through cell divisions, starting from a single cell, the fertilized egg. Therefore, during animal development, a precise regulation of cell division processes is critical not only to produce a large number of cells but also to generate a variety of cell types. Asymmetric cell division is a widespread mechanism for generating cell diversity [1]. During an asymmetric cell division, daughter cells inherit different cellular components (proteins, RNAs, organelles) and thereby have divergent fates. The basic molecular mechanisms of asymmetric cell division in animals have been derived from studies of two model systems: *Drosophila melanogaster* and *Caenorhabditis elegans* [2]. The evolutionarily conserved partitioning-defective (Par) proteins localize asymmetrically along a polarity axis, and control spindle orientation and asymmetric localization of cell fate determinants. Asymmetric cell division requires a high level of coordination between spatial and temporal events. The spatial coordination of spindle orientation with the polarity axis ensures that cell fate determinants are inherited by only one of the two daughter cells. It is still unclear how asymmetric cell division is coordinated in time with other events of the cell cycle. Do cell polarity and cell cycle crosstalk? Are there surveillance mechanisms that ensure that a cell divides only when polarity is established?

Over the past few years, several kinases that play an essential or prominent role in driving cell cycle progression, such as cyclin/cyclin-dependent kinase (Cdk) complexes and the mitotic kinases Polo and Aurora A, emerged as key regulators of cell fate and cell polarity. Conversely, proteins that play a fundamental role in cell polarity have been shown to influence cell cycle progression. Here, we review the current knowledge of the coupling of cell polarity, cell fate and cell cycle progression in *C. elegans* and *D. melanogaster*. We introduce the basic principles and components regulating cell cycle progression with a particular emphasis on components also playing roles in cell polarity and cell fate. We then discuss their role in *C. elegans* and *D. melanogaster* asymmetric cell divisions.

For complementary information, we refer the readers to recent reviews directly focusing on cell polarity and/or cell cycle progression [3–8].

## 3. Principles and components regulating cell cycle progression

**Figure 1.**
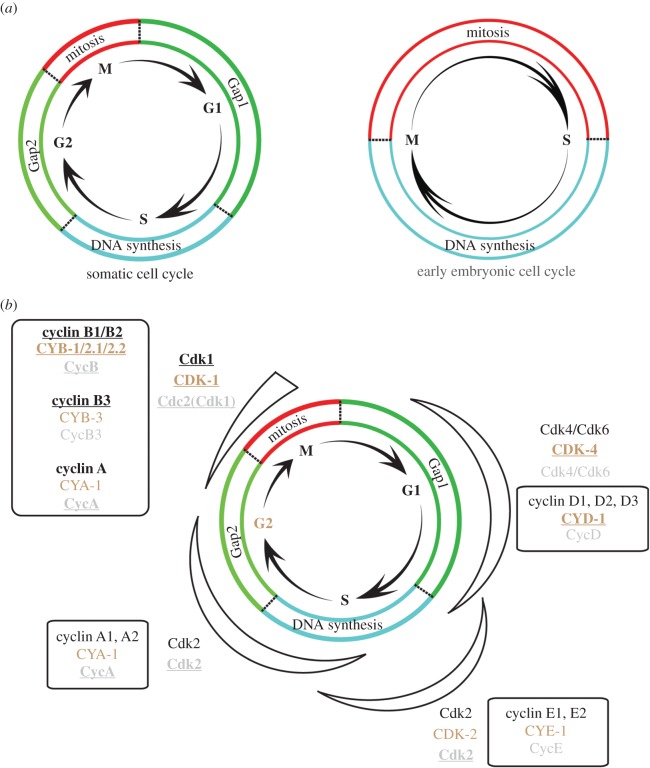
Somatic and embryonic cell cycles and their regulation by cyclin/Cdk complexes. (*a*) Schematic of the cell cycle in somatic and early embryonic cells. DNA synthesis (S, blue), mitosis (M, red) and gap phases (G1, G2, green) are indicated. (*b*) Representation of the cyclin/Cdk complexes regulating the transition between cell cycle phases. The half moons outside the cell cycle represent the level of activity of the indicated complexes (based on studies performed in mammalian cells). Mammalian (black), *Caenorhabditis elegans* (brown) and *Drosophila melanogaster* (grey) homologues are shown. Components underlined and in bold indicate essential players for each model system (adapted from [9]).

Regulation of the cell cycle is critical for the normal development of multicellular organisms. During canonical cell divisions, the cell cycle consists of four distinct phases: G1 (Gap1), S (DNA synthesis), G2 (Gap2) and M phase (mitosis; figure 1*a*). The genetic material is replicated during S phase and segregated into the two resulting daughter cells during M phase. The intervening gap or preparation phases correspond to phases during which a cell grows and gets ready for a new round of DNA synthesis (G1) and prepares mitosis (G2). During early embryonic divisions, S and M phase generally alternate without gap phases (figure 1*a*). Initiation of each phase of the cell cycle is dependent on the proper progression and completion of the previous one, ensuring a unidirectional progression through the cell cycle. How the progression through the phases of the cell cycle is achieved is still under investigation [10–12] but requires cyclin/Cdk complexes assisted by several protein kinases, including the mitotic kinases Polo and Aurora.

### 3.1. Cyclin/Cdk complexes

Work in various organisms has identified a family of conserved heterodimeric serine/threonine kinases made of a regulatory subunit, known as cyclins, and a catalytic component designated as Cdk, as master regulator of the cell cycle [13]. This family of protein kinases orchestrates and drives transitions between the different phases of the cell cycle by phosphorylating key target proteins. In higher eukaryotes, multiple Cdks and cyclins exist, but only five of the standard Cdks (Cdk1, 2, 3, 4, 6) are usually associated with cell cycle control [6]. Cdk1 is activated by A- and B-type cyclins, Cdk2 by E- and A-type cyclins, Cdk3 by C-type cyclins, and Cdk4 and Cdk6 by D-type cyclins.

It was originally thought that Cdks regulate different cell cycle transitions by binding to specific cyclins. However, work in fission yeast has shown that modulating the levels of one cyclin/cdk complex (cyclin B/Cdk1) is sufficient to drive orderly cell cycle transition [10]. Furthermore, mouse knockout experiments targeting cyclins and Cdk loci have revealed that, as in yeast cells, Cdk1 is the only essential Cdk, whereas cyclin A and cyclin B are the only essential cyclins [14–18]. In *D. melanogaster*, Cdk1 (Cdc2) and Cdk2, as well as their cyclin partners cyclin A, B and E, are all required for survival. Cyclin E/Cdk2 complex is required for S phase [19], and cyclin A and B/Cdk1 complexes regulate M phase (figure 1*b*) [20,21]. Although Cdk4 and cyclin D are dispensable for cell proliferation, they are required for cell growth [22]. In *C. elegans,* CDK-1, CDK-2 and CDK-4, and their associated cyclins (CYB-1/3, CYE-1 and CYD-1, respectively), are all essential for viability [23,24].

Although cyclins are indispensable for the catalytic activity of their cognate Cdks and to provide substrate specificity, optimal kinase activity requires additional steps, including the phosphorylation of a key threonine residue located within the activating segment, also known as T-loop, of the Cdk subunit [25]. Beyond phosphorylation of the T loop, Cdk1 is regulated by inhibitory phosphorylation of conserved residues within the active site by Myt1 and Wee1 kinases [26,27]. The Cdc25 dual specificity phosphatase family members reverse these phosphorylation events and thereby activate cyclin B/Cdk1 complex to promote mitotic entry [28].

In summary, cyclin/Cdk complexes are master regulators of cell cycle transitions. Although in mammalian cells there is some functional redundancy between cyclin/Cdk complexes, with Cdk1 being the only essential Cdk, in flies and worms there is a functional specialization of Cdk complexes. As discussed below, some cyclin/Cdk complexes also have a role in polarity regulation, which may explain this functional specialization.

### 3.2. Polo and polo-like kinases

Polo-like kinases (Plks) are critical regulators of mitotic progression. The *polo* gene was discovered more than 20 years ago in *D. melanogaster* and was later found to encode a kinase highly conserved from yeast to man [29–32]. Although *Saccharomyces cerevisiae* (Cdc5) and *Schizosaccharomyces pombe* (Plo1) each have a single Plk that regulates mitotic entry, exit and cytokinesis, metazoans have a minimum of two Plks with different functions. However, Polo (*D. melanogaster*), Plk1 (mammals) and PLK-1 (*C. elegans*) are the closest homologues of Cdc5 and Plo1, and fulfil similar roles during cell division. All Plks share a similar domain organization, with an amino-terminal serine/threonine kinase domain followed by a carboxy-terminal Polo-box domain (PBD). The PBD contains two motifs (Polo box) that form a binding pocket for phosphorylated peptides in target proteins [33]. The priming phosphorylation of target proteins is often provided by Cdks. This mechanism ensures targeted substrate recognition and recruitment of Plk1 to specific sites in the cell, and illustrates how Cdks direct spatio-temporal control of Plks.

Plk1 is activated by phosphorylation of a critical residue in the T-loop by Aurora (A/B) kinases [29]. In mammalian cells, this event is catalysed by Bora, which may help open Plk1 and thereby expose the T-loop to Aurora kinases [34,35].

Plk1 promotes entry into M-phase by activating the Cdc25 phosphatase [36], and by negatively regulating Myt1 [37] and Wee1 kinases [38].

Plk1 is therefore a part of a positive feedback loop that irreversibly activates the cyclin B/Cdk1 complex. Plk1 also responds to polarity to drive mitotic entry and controls polarity in asymmetrically dividing cells (see below).

### 3.3. Aurora kinases

Aurora kinases belong to another family of conserved serine/threonine kinases with a crucial role in mitosis. As for Polo, *S. cerevisiae* and *S. pombe* each contain only one Aurora kinase—Ipl1 (increase in ploidy) and Aurora-related kinase 1 (Ark1), respectively—whereas the mammalian genome contains three Aurora kinases: A, B and C [39]. *S. cerevisiae* Ipl1 was the first member to be described [40]. Later on, the Ipl1 homologue was identified in *D. melanogaster* in a screen for mutations affecting the poles of the mitotic spindle and named Aurora A (referring to aurora borealis) [41].

Although very close in protein sequence and structure (70% identity in the catalytic domain), Aurora A and B have distinct localizations and functions during mitosis. Aurora B is a component of the chromosomal passenger complex (with INCENP, survivin and borealin), and is essential for chromosome segregation and cytokinesis. Consistent with its function, Aurora B localizes at centromeres in prophase and metaphase, at the cortex and spindle midzone in anaphase, and at the midbody in telophase [42]. Aurora C has a similar localization but is specifically expressed in germ cells of mammals and has not been found in other organisms [39]. Aurora A localizes at centrosomes and at the spindle poles, and is required for mitotic entry, centrosomes maturation and spindle formation. In mammals, Aurora A promotes mitotic entry by phosphorylating and activating CDC25B [43,44], and by promoting Plk1 activation via the cofactor Bora [34,35].

Activation of Aurora kinases occurs by autophosphorylation of the T-loop, and is promoted by the interaction with cofactors such as the microtubule-associated protein TPX2 for Aurora A and INCENP for Aurora B [45]. A single amino acid substitution, which changes cofactor affinity, transforms Aurora A into Aurora B [46,47], indicating that interaction with different partners is essential to specify the localization and function of these kinases during mitosis. Aurora B and C do not have established roles in polarity. Although Aurora B activates Polo in flies [48], this function appears specific for centromeric Polo and does not affect polarity. By contrast, Aurora A has an established role in polarity in *D. melanogaster*, *C. elegans* and mammalian cells (see below).

## 4. Coupling cell polarity and cell cycle progression in *Caenorhabditis elegans*

### 4.1. Anterior–posterior polarity and asynchronous mitotic entry

The early *C. elegans* embryo is an attractive model system for studying the mechanisms coupling cell polarity and cell cycle timing regulation [7,49]. Like *X. laevis* and *D. melanogaster*, the embryonic cell division cycles in *C. elegans* consist of rapid phases of DNA replication alternating with mitosis, without intervening gap phases (figure 1*a*). However, in contrast to *Xenopus laevis* or *D. melanogaster*, the early *C. elegans* embryo undergoes a series of asymmetric and asynchronous divisions to produce five somatic founder cells (AB, E, MS, C and D) and the primordial germ cell (P4) [50]. The generation of these precursor cells requires a precise coupling between cell polarity and the cell cycle, starting from the first asymmetric cell division, which generates two blastomeres of different sizes and developmental potentials that divide asynchronously. The anterior larger AB blastomere, which is the precursor of the somatic lineage, enters into mitosis 2 min before the posterior P1 blastomere, which will give origin to the germline and to somatic cells. The cell cycle asynchrony is highly reproducible and regulated by anterior–posterior (A–P) polarity. However, its precise role for embryonic development is not fully understood, as mutations that affect the asynchrony also result in other defects during embryonic development.

**Figure 2.**
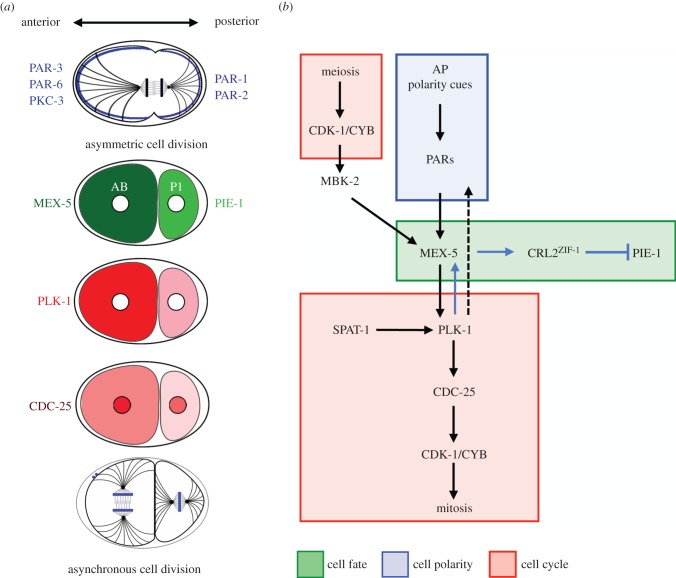
Coordination of cell polarity, cell fate and cell cycle progression in the early *Caenorhabditis elegans* embryo. (*a*) The top drawing shows a schematic of a one-cell embryo in mitosis (metaphase) with PAR-3, -6 and PKC-3 (blue) at the anterior, and PAR-1 and PAR-2 (blue), at the posterior cortex. The lower drawings are schematic of two-cell embryos showing the localization and levels of MEX-5 (dark green) and PIE-1 (light green), and the cell cycle regulators PLK-1 (red) and CDC-25 (red). The first asymmetric cell division generates a large anterior blastomere AB, which divides before the smaller posterior P1 blastomere. (*b*) A–P polarity cues and PAR proteins control the asymmetric localization of MEX-5, which directs PLK-1 localization (as indicated by arrows). SPAT-1 activates PLK-1 to promote earlier mitotic entry in the anterior blastomere. In turn, PLK-1 phosphorylates and activates MEX-5 (blue arrow). PLK-1 may also control polarity directly, by phosphorylating PAR proteins (PARs; dotted arrow). MEX-5 promotes proteasomal degradation of PIE-1 by CRL2^ZIF-1^ in the somatic lineage (blue arrow). Red boxes indicate proteins with well-established function in cell cycle regulation, blue boxes indicate polarity proteins and green boxes indicate cell fate determinants, in this and other figures. Positive regulation indicated by arrows, negative regulation indicated by bars.

A–P polarity of the embryo is under the regulation of PAR proteins: PAR-1 to PAR-6 and atypical protein kinase C (PKC-3) [51,52]. Molecularly, PAR proteins are quite divergent: PAR-1, PAR-4 and PKC-3 are protein kinases, PAR-2 is a ring-finger protein, PAR-3 and PAR-6 are PDZ-domain proteins, whereas PAR-5 is a 14-3-3 protein [53]. PAR-1, -2, -3, -6 and PKC-3 localize asymmetrically in the one-cell embryo (figure 2*a*). With the exception of PAR-2, PAR proteins are highly conserved across species.

The polarization of the one-cell embryo is a highly dynamic process and proceeds in two distinct phases: establishment and maintenance [54]. Just after fertilization, the embryo is not polarized. PAR-3/PAR-6/PKC-3 (anterior PAR proteins) localize uniformly at the cortex, PAR-1/PAR-2 (posterior PAR proteins) are in the cytoplasm, and the embryonic cortex is highly contractile and under tension [55]. Polarity establishment is triggered by a signal from the sperm-donated centrosome, which breaks the initial symmetry by downregulating cortical contractility at the site of sperm entry (the future posterior pole), and thus results in the displacement of PAR-3/PAR-6/PKC-3 from the posterior to the anterior cortex and allows PAR-1 and PAR-2 to localize to the posterior (establishment phase) [54]. Then, mutual inhibition between anterior and posterior PAR proteins maintains the two domains (maintenance phase) [51,52]. This mutual inhibition depends, in part, on reciprocal phosphorylation events that prevent cortical localization. The posterior kinase PAR-1 phosphorylates PAR-3, inhibiting its cortical localization at the posterior. The anterior kinase PKC-3 phosphorylates PAR-1 and PAR-2 to exclude them from the anterior [56,57]. In the absence of the anterior PAR proteins, the posterior PAR proteins occupy the entire embryonic cortex. Vice versa, in the absence of the posterior PAR proteins, anterior PAR proteins occupy the entire cortex. Accordingly, reducing the levels or activity of the anterior PAR complex suppresses *par-2* loss-of-function phenotypes [58–61].

Once localized in reciprocal domains, PAR proteins dictate all the asymmetries in the early embryo, including asymmetric spindle positioning, cytoplasmic protein localization and asynchronous mitotic entry at the two-cell stage (figure 2*a*) [51,52].

### 4.2. PAR proteins act via MEX-5/6 and polo-like kinase to regulate cell cycle progression

MEX-5 and MEX-6 (muscle in excess) are two nearly identical and partially redundant cytoplasmic zinc-finger RNA binding proteins that become distributed in a cytoplasmic gradient along the A–P axis of the embryo [62]. Because of the redundancy in function, we will often refer to them as MEX-5/6.

How the cytoplasmic gradient is established has been shown mainly for MEX-5. MEX-5 anterior enrichment is the result of an underlying gradient of MEX-5 diffusivity, which depends on the PAR-1 kinase [63–65]. PAR-1 phosphorylates MEX-5, and thereby stimulates its release from slow-diffusive, RNA-containing complexes in the posterior cytoplasm. MEX-5 phosphorylation is antagonized by the spatially uniform PP2A phosphatase. Localized posterior phosphorylation and uniform dephosphorylation reactions are sufficient to generate a stable concentration gradient of MEX-5 in the cytoplasm [64].

MEX-5/6 act as polarity transducers and are crucial for establishing soma/germline asymmetry [62]. MEX-5/6 act, at least in part, by activating the CUL-2-based E3-ligase using the substrate-recognition subunit ZIF-1 (cullin ring E3 ligase, CRL2^ZIF-1^) to target several germplasm proteins for degradation in the somatic lineage (figure 2*b*) [66].

MEX-5/6 also regulate the timing of division by binding to PLK-1 and promoting its enrichment in the anterior cytoplasm of the one-cell embryo [67]. This leads to higher levels of PLK-1 in the AB cell compared with P1 in two-cell embryos, and PLK-1 drives earlier mitotic entry of AB (figure 2*b*) [68–70]. How do MEX-5/6 anchor PLK-1 in the anterior? The minibrain kinase, MBK-2, which is activated at the end of meiosis II by CDK-1 (figure 2*b*) [71], phosphorylates MEX-5 on a polo-docking site [67]. Once phosphorylated, MEX-5 interacts with the PLK-1-PBD and thereby anchors PLK-1 in the anterior part of the embryo. The interaction between MEX-5 and the PLK-1 PBD may also contribute to PLK-1 activation, possibly by releasing the intra-molecular interaction between the kinase domain and the PLK-1 PBD [72].

What happens during the following asymmetric cell divisions of the P lineage? The daughters of P1, EMS (anterior daughter) and P2 (posterior daughter), divide asynchronously with EMS always entering mitosis before P2. Interestingly, PLK-1 is present in higher levels in EMS compared with P2 (A.N., N.T., M.G. & L.P. 2010, unpublished data). However, the mechanism of this enrichment and whether this is responsible for earlier mitotic entry has not been investigated.

In addition to PLK-1, CDC-25.1 (one of the Cdc25 isoforms) is another important cell cycle regulator, exhibiting an asymmetry in protein levels in the early embryo. CDC-25.1 is specifically enriched in the AB nucleus when compared with P1. Fluorescence recovery after photobleaching experiments revealed that the rate of nuclear accumulation of GFP::CDC-25.1 is higher in AB than P1 and depends on PLK-1 activity [70]. These observations suggest a model in which higher levels of anterior PLK-1 induce higher levels of nuclear CDC-25.1 in the AB compared with the P1 cell, which promotes earlier mitotic entry in the AB blastomere (figure 2*a*). However, the precise mechanisms by which PLK-1 regulates CDC-25.1 nuclear localization are not understood.

Although the PAR network controls PLK-1 and CDC-25.1 localization via MEX-5/6, it may also regulate these proteins more directly, possibly via the PAR-4 kinase. PAR-4 is homologous to LKB1, a human kinase associated with Peutz–Jeghers syndrome [73,74]. PAR-4 is required for several developmental processes in the early *C. elegans* embryo and, notably, for the establishment of cytoplasmic asymmetries and cell cycle regulation [75]. Depletion of PAR-4 results in an asymmetric cell division, but with the two resulting blastomeres dividing synchronously [76]. Although PAR-4 and PAR-1 similarly regulate PLK-1 asymmetric localization, *par-4* mutants have abnormally high levels of nuclear CDC-25.1 [70]. These observations suggest that PAR-4 may have additional roles that result in inhibition of CDC-25.1 nuclear localization, perhaps by regulating PLK-1 activity. Consistent with this hypothesis, partial inactivation of *cdc-25.1* or *plk-1* suppresses *par-4* mutant lethality [58].

To summarize, PAR proteins regulate cell cycle timing in the two-cell embryo by controlling the localization of key cell cycle components via MEX-5/6.

### 4.3. The DNA replication checkpoint pathway regulates AB/P1 asynchrony in the early embryo

In wild-type embryos, the PLK-1 pathway accounts for 60% of the cell cycle delay between AB and P1 cells [68]. A second pathway downstream of PAR polarity that regulates 40% of the asynchrony of cell division engages the DNA replication checkpoint [77]. This pathway, which involves the kinases ataxia–telangectasia mutated related ATR (ATL-1 in *C. elegans*) and checkpoint kinase one (CHK-1), is primarily activated in response to defects in DNA replication to ensure that mitosis is not initiated until the DNA is fully replicated [78]. The finding that this checkpoint pathway regulates AB/P1 asynchrony originally came from the observation that interfering with DNA replication delays interphase in AB and P1 blastomeres, with P1 being considerably more affected than AB. This leads to the formation of three-cell-stage blastomeres that are never observed in wild-type [79,80]. Inactivation of ATL-1 or CHK-1 suppresses this phenotype and restores normal cell cycle timing in these DNA replication mutants [77].

How and why the checkpoint is preferentially activated in the P1 blastomere remains largely unknown. The DNA replication checkpoint has been investigated so far in artificial conditions that block DNA replication using hydroxyurea. In two-cell embryos, the checkpoint is activated in a developmental non-artificial context, and this activation is controlled by polarity cues. PAR proteins might regulate the asymmetric localization of checkpoint components, or might differentially control DNA replication in the two blastomeres. Alternatively, one or several DNA replication factors might be present in limited quantity in the P1 blastomere as a consequence of the smaller size [81]. However, embryos are probably fully loaded with high levels of DNA replication factors required for rapid S phase during early embryonic development. Furthermore, *par-4* mutant embryos divide asymmetrically, but AB and P1 cells enter mitosis synchronously [76], suggesting that the cell size asymmetry might not be causing DNA replication checkpoint activation. In the future, it will be critical to develop assays to analyse the temporal programme of DNA replication between AB and P1 blastomeres to determine whether checkpoint activation is the result of differential DNA replication programmes, or whether some checkpoint components are directly activated by PAR proteins, such as PAR-1 or PAR-4.

In summary, A–P polarity cues control cell cycle duration in two-cell-stage *C. elegans* embryos through two mechanisms. First, they ensure the accumulation of PLK-1 in AB via the cell fate determinant MEX-5; second, they contribute to the preferential ATL-1 and CHK-1 activation in P1 by still unknown mechanism. Together, these two pathways couple A–P polarity cues with cell cycle progression during early embryonic development.

### 4.4. Cell cycle components regulate polarity in the early *Caenorhabditis elegans* embryo

Although polarity proteins regulate cell cycle timing by controlling the differential localization of core cell cycle components in early embryos, cell cycle factors conversely regulate several aspects of embryonic polarity. In early embryos, the cyclin E (CYE-1)/CDK-2 complex controls polarity establishment by regulating centrosome maturation [24]. The sperm provides a pair of centrioles that is incapable of polarizing the cortex [82,83]. CYE-1/CDK-2 promotes the centrosomal recruitment of several proteins required for polarity establishment, such as SPD-2 and SPD-5, thus contributing to centrosome maturation and polarity establishment [24]. Notably, this effect appears independent of its canonical role in DNA replication and cell cycle progression, because inhibiting DNA replication or delaying the cell cycle by depleting other essential cyclins does not affect establishment of polarity [24].

Other cell cycle components that regulate polarity were identified in a genome-wide *par-2 temperature-sensitive* (*ts*) suppressor screen, and include the B-type cyclins CYB-2.1 and CYB-2.2 and the Bora homologue SPAT-1 [60].

SPAT-1 physically interacts with PLK-1, and embryos depleted of SPAT-1 or PLK-1 present similar cell cycle and polarity defects consistent with SPAT-1 acting as a PLK-1 activator [84]. Similar to SPAT-1 depletion, inactivation of PLK-1 suppresses *par-2ts*-associated lethality and polarity defects, whereas loss of SPAT-1 or PLK-1 results in polarity defects [84]. PLK-1 effect on polarity is unlikely to be due to the cell cycle delay, as slowing down the cell cycle with other means does not result in polarity defects, nor in *par-2ts* mutant rescue [24,84]. These observations suggest that PLK-1 may play an active role in polarity, possibly by phosphorylating PAR proteins. Alternatively, PLK-1 may control polarity by regulating the actomyosin cytoskeleton, which is essential for polarity establishment and maintenance [51]. It is not known how the B-type cyclins regulate polarity, but they could do so by controlling the activity of SPAT-1 or PLK-1 via a Cdk.

Aurora A kinase (AIR-1) is another cell cycle component involved in cell polarity. Loss of *air-1* results in centrosome maturation defects, cell cycle delay and polarity defects [84–87]. AIR-1 might control polarity indirectly by regulating centrosome maturation [86] and/or by activating PLK-1 [84]. Indeed, in mammalian cells, Aurora A phosphorylates and activates Plk1 [34,35]. However, in *C. elegans*, there is no evidence for AIR-1 to be involved in PLK-1 activation [84]. Alternatively, AIR-1 could control polarity more directly by phosphorylating polarity components, as is the case in *D. melanogaster*, where Aurora A phosphorylates Par6 and aPKC [88,89], or in mammalian neurons, where Aurora A phosphorylates Par3 [90].

Although available data implicate cyclin/Cdk complexes, PLK-1 and AIR-1 kinases in the control of cell polarity, further work is required to identify their critical targets, and to better understand the coupling between cell cycle and cell polarity during *C. elegans* embryonic development.

## 5. Coupling cell polarity, cell fate and cell cycle progression in *Drosophila melanogaster*

### 5.1. Par1 regulates cyclin A during asymmetric divisions of the male germline stem cell

**Figure 3.**
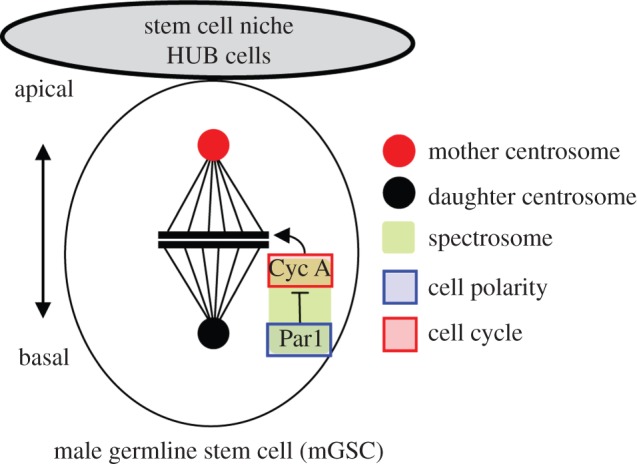
Par1 regulates the asymmetric cell division of the male germ stem cell in *Drosophila melanogaster.* A cluster of supporting cells, called the HUB cells (in light grey), provides the niche that is essential for the maintenance of stem cell identity of the male germline stem cell. The mitotic spindle aligns towards the HUB cells, generating one daughter that remains close to the HUB cells and maintains stem cell identity, and one daughter far away from the HUB, which differentiates. Par1 (blue) and cyclin A (CycA, red) are part of a surveillance mechanism that localizes to the spectrosome (green) and prevents mitotic entry until proper orientation of the mitotic spindle is achieved.

The asymmetric division of male germline stem cells (mGSCs) in *D. melanogaster* offers an example of coupling between polarity and cell cycle progression. In the male germline, the HUB cells constitute a stem cell niche that provides signals to maintain stem cell identity (figure 3). During the division of mGSCs, the mother centrosome remains positioned close to the HUB cells, whereas the daughter centrosome migrates to the opposite side [91,92]. This centrosome orientation pattern results in a division that maintains one daughter within the niche and displaces away from the niche the daughter that differentiates, leading to an asymmetric outcome of the stem cell division (figure 3). Proper centrosome positioning and spindle orientation perpendicular to the niche is thus critical to maintain germ stem cell homeostasis. The polarity protein Par1, together with cyclin A (CycA), is part of a surveillance mechanism ensuring that mitosis is not initiated until the centrosomes have reached their correct position [93,94]. CycA localizes to the spectrosome, an endoplasmic reticulum- and cytoskeleton-like structure [95], in a Par1-dependent manner, and this localization is essential to keep a functional checkpoint. When CycA spectrosomal localization is impaired, for instance, in *par1* mutants, or following the expression of a CycA mutant that cannot localize to the spectrosome, the checkpoint is not functional, resulting in centrosome positioning defects [94]. Although it is not known how Par1 controls CycA localization, nor how CycA controls the checkpoint, this example illustrates how a well-defined polarity protein couples centrosome positioning, spindle orientation and cell cycle progression to maintain stem cell identity.

### 5.2. Prospero limits cell proliferation in ganglion mother cell

**Figure 4.**
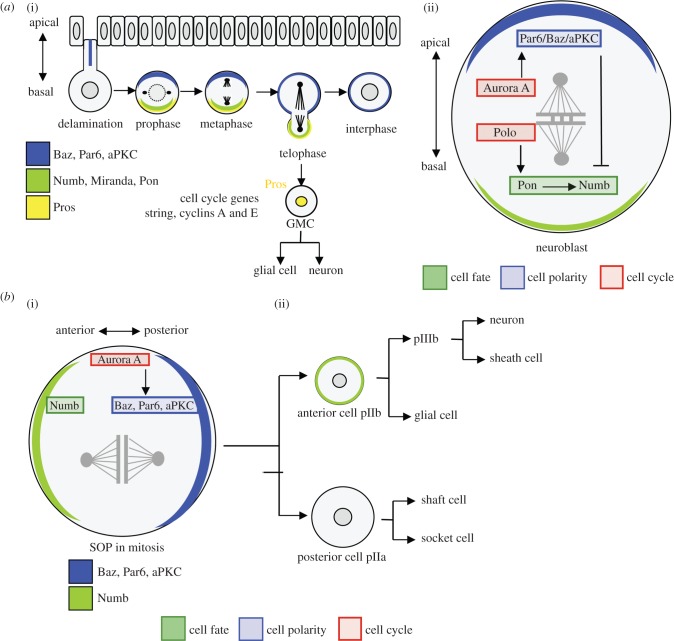
Asymmetric cell division of *Drosophila melanogaster* neuroblasts (NB) and sensory organ precursor (SOP) cells. (*a*) (i) After delamination from the layer of the epithelium, the NB divides asymmetrically to generate a new NB and a ganglion mother cell (GMC). Prospero (Pros, yellow) localizes as a crescent on the basal side of the NB and it is segregated to the GMC, where it accumulates in the nucleus. Nuclear Pros prevents the expression of cell cycle genes contributing to terminal differentiation of the GMC, which divides once to generate neuron and a glial cell. (ii) Schematic representation of the molecular links between cell cycle components (Polo, Aurora A) and cell fate determinants that contribute to the neuroblast asymmetric cell division. (*b*) Asymmetric division of the sensory organ precursor (SOP) cell of the *Drosophila* PNS, which gives rise to an anterior cell, pIIb and a posterior cell, pIIa. (i) Schematic of the link between Aurora A and Numb that contributes to the SOP asymmetric cell division. Numb (green) accumulates in the pIIB cell. pIIa generates one shaft cell and one socket cell, whereas pIIb generates a glial cell and pIIIb that produces a neuron and a sheat cell. Numb accumulates in pIIb and selectively segregates in some further progenies. (ii) Schematic of the link between Aurora A and Numb that contributes to the SOP asymmetric cell division.

During brain development, a large number of cell types are generated through asymmetric cell divisions. In *Drosophila*, neuroblasts (NBs) and sensory organ precursor cells (SOPs) in the central or peripheral nervous system (CNS or PNS, respectively) have emerged as key model systems to study asymmetric cell division, and have provided crucial insights into the mechanisms coupling polarity and cell cycle machineries (figure 4).

During embryogenesis, NBs delaminate from the epithelial monolayer and enter mitosis (figure 4*a* (i)) [96,97]. The apical–basal polarity of an epithelial cell is governed by the Par3 (bazooka/Baz), Par6 and aPKC complex, and is inherited by the delaminating neuroblast. During prophase, the apically localized Par complex directs the basal distribution of neural cell fate determinants such that upon asymmetric cell division, NBs generate two daughter cells of different identities: a bigger apical neuroblast and a smaller ganglion mother cell (GMC; figure 4*a* (i)). These two daughter cells have different fates: the neuroblast divides in the same asymmetric manner several times, whereas the GMC divides only once, and differentiates into neurons and/or glial cells [1,98]. The gene expression pattern of neuroblasts changes in the course of their life, so that the early-born neuroblasts express different genes than the ones born later [99]. The expression of these genes is inherited and maintained by the GMC, and therefore NBs produce different neurons and glia during development.

The transcriptional regulator Prospero (Pros) is one of the cell fate determinants that segregates into the GMC [100]. Upon completion of mitosis, Pros translocates from the cytoplasm into the nucleus of the GMC where it activates or represses specific genes to specify the GMC fate (figure 4*a* (i)) [101,102]. In *pros* mutants, string/Cdc25, cyclin A and cyclin E are ectopically expressed and cells are mitotically active, whereas when Pros is overexpressed, these cell cycle genes are repressed. Thus, segregation of Pros to the GMC limits its mitotic potential and triggers differentiation. Nevertheless, depending on the developmental context, Pros can also promote, instead of limiting, proliferation. In the lineage, producing longitudinal glia Pros promotes cell proliferation by positively regulating cyclin E [103], suggesting that additional factors can modulate Pros function depending on the biological context.

In summary, studies in fly neuroblasts highlighted that polarity regulates the segregation of Prospero to the GMC, and this in turn limits the mitotic potential of the GMCs.

### 5.3. Mitotic kinases, cyclin/Cdks complexes and their role in asymmetric cell division

The asymmetric localization of cell fate determinants in neuroblasts is dynamic and timely regulated during the cell cycle. During late prophase/early metaphase, Pros, Numb (Notch signalling inhibitor) and their adaptor proteins, Miranda (Mira) and partner of Numb (Pon), localize as crescents at the basal NB cortex (figure 4*a* (ii)).

How is such spatio-temporal organization executed? Cyclin/Cdks and mitotic kinases are implicated in this coordination. NBs in which Cdk1 (Cdc2) function is attenuated without arresting mitosis establish apical polarity in interphase, but are unable to maintain it during mitosis [104]. How Cdc2 dictates NB asymmetric division is not understood, but Cdc2 may phosphorylate one or several apical cell fate determinants in mitosis to maintain cortical polarity.

Aurora A, which in flies is regulated by Bora [105], controls the localization of aPKC and Numb during mitosis in both NBs and SOPs [88,89,106,107]. SOPs, the progenitors of PNS, exhibit A–P polarity and divide asymmetrically, giving rise to two different precursor cells: a smaller anterior cell (pIIb) and a bigger posterior cell (pIIa; figure 4*b*). Subsequently, pIIb will give rise to a neuron and a sheath cell, whereas pIIa gives rise to one hair and one socket cell. The specific developmental programmes for pIIb and pIIa cells are triggered by the differential Notch signalling present in both cells, which is executed by the asymmetric distribution of Numb, the Notch inhibitor [108]. Although Notch is present in both cells, Numb targets the transmembrane Notch receptor to endocytosis in pIIb, thus repressing the signalling cascade [106].

Aurora A directs Numb localization indirectly via timely regulation of the composition of the Par complex in NBs and SOP cells [89]. In interphase, the Par complex is composed of aPKC, Par6 and Lgl (the tumour suppressor lethal giant larvae). At mitosis onset, Aurora A phosphorylates Par6 and thus triggers rearrangement of the Par complex. Following Par6 phosphorylation, aPKC becomes more active and phosphorylates Lgl [109], which then dissociates from the cortex and localizes to the cytoplasm. Dissociation of Lgl allows Baz to interact with aPKC/Par6, and this confers novel substrate specificity to the Par complex. Baz recruits Numb, which is then phosphorylated by aPKC. aPKC phosphorylation of Numb has two functions: it excludes Numb from the cortex where aPKC is present (figure 4*b*) [89,110] and reduces its endocytic activity required for the repression of Notch signalling in the pIIb cell [111]. Loss of *aurora A* in SOPs results in Numb accumulation around the entire cortex and Numb segregation into both daughter cells, which adopt the same fate and give rise to two hairs and two socket cells without neuron and sheath cells [106].

In *aurora A* mutant NBs, aPKC and Numb are segregated into both daughter cells, where aPKC phosphorylates Numb and thus reduces its activity [111]. Owing to low Numb activity, daughter cells are transformed into neuroblasts, leading to NB overproliferation and eventual tumour formation [89].

Polo is another regulator of the G2-M transition that suppresses tumour formation by controlling the localization of Numb. In *polo* mutants, aPKC, Pon and Numb are not asymmetrically localized, resulting in abnormal divisions with the production of supplementary neuroblasts at the expense of neurons [112]. Wang *et al*. showed that Polo phosphorylates Pon and thus directs Pon basal localization [112]. Pon, in turn, localizes Numb (figure 4*a*). Therefore, Polo acts as a tumour suppressor by controlling the localization of cell fate determinants and promoting differentiation.

Loss of both *polo* and *aurora A* results in Numb mislocalization. Aurora A activates Plk1 in mammalian cells [34,35], whereas Aurora B has been shown to activate centromeric Polo in flies [48]. If Aurora A activates Polo in *D. melanogaster* as well, this may suggest the existence of an additional mechanism in which Aurora A regulates Numb localization by activating Polo, which in turn controls Pon.

Therefore, in NBs and SOPs, the link between cell cycle and asymmetric cell division is provided by the mitotic kinases, which regulate the localization of cell fate determinants.

### 5.4. Cyclin E regulates the fate of neuroblasts

Cyclin E is important for polarity establishment in the *C. elegans* embryo [24]. In flies, cyclin E (CycE) is required for fate specification in the NB6-4 lineage of neuroblasts [113]. NB6-4 thoracic neuroblasts (NB6-4t) divide asymmetrically and give rise to neuronablast and GMC, which ultimately produce five to six neurons and three glial cells. Conversely, NB6-4 abdominal neuroblasts (NB6-4a) undergo a symmetric division and generate two glial cells [114]. This fate difference between thoracic and abdominal neuroblast is due to CycE. CycE is expressed in NB6-4t neuroblasts where it promotes asymmetric division. Notably, the role of CycE in cell fate determination is independent of its role in cell cycle progression but involves the regulation of the localization and activity of Pros [113,115]. In NB6-4t neuroblasts, CycE binds to Pros and facilitates its cortical asymmetric localization. During cell division, Pros is segregated to the glial-producing daughter cell where it translocates to the nucleus and repress genes required for self-renewal [101,102]. After NB6-4t division, CycE is only detected in the neuronal and not the glial progenitor. The lack of CycE results in NB6-4t-to-NB6-4a transformation where nuclear Pros is present in all cells, leading to the production of only glial cells. On the other hand, ectopic expression of CycE in the NB6-4a lineage causes its transformation to NB6-4t, where only one progeny inherits Pros, and neurons are produced in addition to glia [113]. Interestingly, Pros represses the expression of CycE, which suggests a negative feedback loop where the result of CycE and Pros antagonistic functions decides whether a cell differentiates or continues dividing. The mechanisms explaining how CycE regulates Pros are not known but may involve the CycE-associated kinase. Cortical Pros is highly phosphorylated, whereas its nuclear fraction is not [116], which raises the possibility that phosphorylation directs Pros localization and inhibits its translational activity in NB6-4t.

CycE function in cell fate appears to apply to other neuroblasts, not only the NB6-4 lineage [115]. Accumulating evidence also indicates that cyclin E plays an important role in cell fate determination by regulating critical cell fate determinants not only in the somatic lineages but also in the germline, where cyclin E promotes self-renewal and prevents meiotic differentiation both in *D. melanogaster* [117] and in *C. elegans* [118,119]. Identifying the critical targets of cyclin E/Cdk2 remains a challenge for the future.

## 6. Concluding remarks

**Figure 5.**
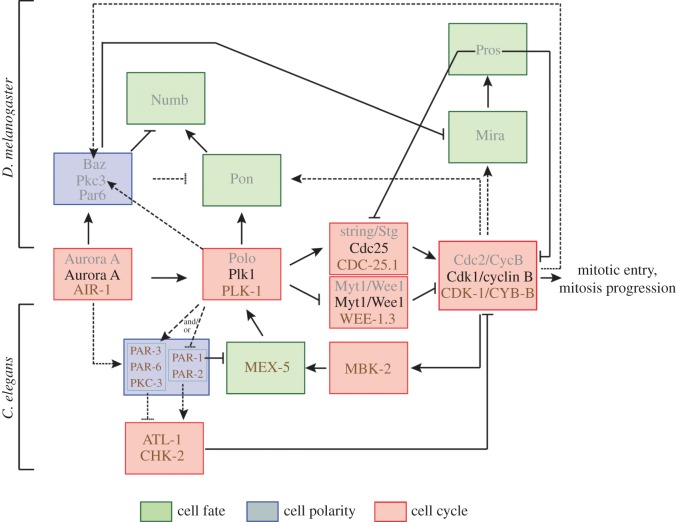
Molecular circuitry linking the cell cycle, cell fate and cell polarity machineries in *Drosophila melanogaster* and *Caenorhabditis elegans*. The pathways involved in *D. melanogaster* are on the top part of the figure, those in *C. elegans* on the bottom part. *Drosophila melanogaster* (grey), *C. elegans* (brown) and mammalian (black) homologues are indicated. Arrows and lines with the bar indicate positive and negative regulation, respectively. Dotted lines mean that the precise molecular mechanism between indicated proteins is not yet well understood; however, the genetic data suggest a crosstalk between these players.

Considerable progress has been made in our understanding of the cell cycle and asymmetric cell division machineries (figure 5). Although it now seems obvious that the two machineries have to be coordinated, the finding that cell cycle components regulate cell polarity and cell fate was fortuitous. The link between the cell cycle and the cell polarity and cell fate machineries was often revealed through genetic screens. For instance, screens for genes regulating asymmetric cell divisions led to the identification of cell cycle components, both in *C. elegans* [60,84,120] and in *D. melanogaster* [104,105]. There is now substantial experimental evidence in the literature giving strong support for more direct hypothesis-driven experiments [112] to further explore the links between the cell cycle and asymmetric cell division machineries.

In both worms and flies, Par1 appears to have a more direct role in controlling cell cycle progression—in *C. elegans* by phosphorylating MEX-5 and controlling its A–P gradient which is, in turn, necessary for the enrichment of PLK-1 at the anterior [64,67], and in *D. melanogaster* by regulating the localization of cyclin A in a yet-unknown manner [94]. It will be interesting to investigate whether other Par proteins also have a direct role in controlling cell cycle regulators.

Protein kinases such as the cell cycle kinases Cdk1, Polo and Aurora A, and the polarity kinases Par1, Par4 and Pkc3, play a prominent role in this coordination. Dissecting their role will require the identification of their numerous substrates. Characterization of the function of these substrates using cell biology, genetic and biochemistry will determine whether and how they coordinate cell cycle progression with cell polarity and asymmetric cell division. For instance, given the number of substrates that Plk1 phosphorylates to regulate mitosis [29], it is tempting to speculate that beyond Pon in *D. melanogaster* and MEX-5 in *C. elegans*, Plk1 phosphorylates and regulates other cell fate determinants, and possibly also the Par proteins, to couple polarity and cell cycle progression.

Here, we have discussed examples of the link between cell polarity and cell cycle progression in two metazoan model systems, which have contributed to our understanding of this process. This link also exists in mammalian cells, where several cell cycle components play an important role in neuronal differentiation. For instance, Aurora A regulates neuronal polarity by phosphorylating Par3 in mouse [90]. Furthermore, during the development of the mouse neocortex, cyclin D2 is differentially distributed upon asymmetric cell division of the radial glial cells such that the daughter cell, which inherits cyclin D2, maintains self-renewal capacity [121,122].

However, we still do not know how these two processes are coordinated. Cell cycle and cell polarity may ‘simply’ be jointly regulated, without crosstalk. Alternatively, surveillance mechanisms such as polarity checkpoints may exist that arrest or delay one process when the other is defective. Further work will be required to understand exactly how these processes are coordinated during development and in adult polarized cells.
